# Electrochemical Detection of Synthetic Vanillin Using a Strontium Pyrophosphate Nanorod-Modified Electrode

**DOI:** 10.1021/acsmaterialsau.4c00165

**Published:** 2025-01-21

**Authors:** Balasubramanian Sriram, Sakthivel Kogularasu, Sea-Fue Wang, Guo-Ping Chang-Chien

**Affiliations:** † Department of Materials and Mineral Resources Engineering, 34877 National Taipei University of Technology, Taipei 106, Taiwan; ‡ Super Micro Mass Research and Technology Center, 63330 Cheng Shiu University, Kaohsiung 833301, Taiwan; § Center for Environmental Toxin and Emerging-Contaminant Research, Cheng Shiu University, Kaohsiung 833301, Taiwan; ∥ Institute of Environmental Toxin and Emerging-Contaminant, Cheng Shiu University, Kaohsiung 833301, Taiwan

**Keywords:** strontium pyrophosphate, Sr_2_P_2_O_7_, synthetic vanillin, food samples, electrochemical sensor

## Abstract

Synthetic vanillin (VLN) is extensively utilized as a flavoring agent in the food and pharmaceutical industries, raising health concerns due to its synthetic origin and widespread consumption. Strontium phosphate (Sr_2_P_2_O_7_) has been a desirable electrode modifier in recent years due to its distinct structural and electrochemical characteristics. Due to its stability, efficacy, and electrocatalytic capabilities, Sr_2_P_2_O_7_ has emerged as a competent electrocatalytic material. This study presents the fabrication and application of a screen-printed carbon electrode (SPCE) modified with Sr_2_P_2_O_7_ nanorods as a sensitive and selective electrochemical sensor for VLN detection. Sr_2_P_2_O_7_ nanorods were synthesized via a sonochemical approach and thoroughly characterized by spectroscopic and electrochemical techniques to confirm their structural and functional properties. Quantification of VLN was achieved using a sensitive amperometric (*i*–*t*) technique, yielding a lower detection limit of 0.52 nM and a wide linear detection range of 0.001–726.8 μM. Additionally, real-sample analyses in food samples exhibited recovery rates (±98.00–99.66%), underscoring the platform’s practical applicability for monitoring synthetic VLN in real-world conditions. This work highlights the potential of Sr_2_P_2_O_7_-modified SPCEs as reliable tools for food safety applications, offering a cost-effective, disposable solution for synthetic vanillin detection.

## Graphical abstract

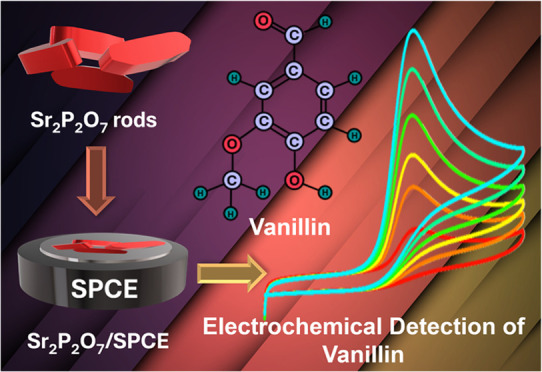

## Introduction

Vanilla, derived from the fruit of a tropical orchid, holds significant importance as a natural aromatic agent.^1^ The principal aromatic compound in vanilla is vanillin (VLN), contributing to its characteristic aroma and flavor. There are more than 200 components in VLN, which is present in cured vanilla pods at quantities of 1.0–2.0 wt %. The primary component responsible for its sensory appeal is 4-hydroxy-3-methoxybenzaldehyde.^2,3^ Throughout the world, VLN is used as an addition in food and as a flavoring in many different products, including sweets, drinks, medicine, and fragrance.^4^ VLN imparts a unique flavor during aging in winemaking, with p-hydroxybenzaldehyde, p-hydroxybenzoic acid, and vanillic acid being the predominant VLN constituents.

Despite annual global production exceeding 12,000 tons, natural VLN accounts for less than 1% due to limited vanilla sources.^5^ To meet demand, 99% of VLN on the market is synthesized through chemical or biochemical methods, making it readily available and cost-effective.^6^ VLN also serves as a precursor in drug synthesis, including L-dopa for Parkinson’s disease treatment, and as a general coloring agent in chemical reaction monitoring.^7–9^ Natural VLN, with its distinctive constituents, differs significantly from synthetic variants, which can lead to adverse health effects, including migraines and aggravation in sensitive individuals. Consequently, regulatory bodies, including the United Nations, have set permissible daily intake limits for VLN at less than 10 mg/kg, as excessive intake can cause symptoms like headaches, nausea, and severe damage to the liver and kidneys.^10^

Multiple analytical techniques have been developed to accurately determine synthetic VLN in food products, including fluorescence,^11^ capillary electrophoresis,^12^ HPLC,^13^ spectrophotometry,^14^ GC-MS,^15^ and LC-MS.^16^ While effective, these methods require specialized operators, are costly, and are time-intensive. In recent years, electrochemical sensors have gained significant attention as alternative methods due to their simplicity, rapid response, online detection capability, and high sensitivity.^17–19^

Current studies have highlighted the potential of metal oxide nanoparticles, including metal phosphates, in electrochemical sensor applications due to their high surface area, electrocatalytic properties, and environmental sustainability.^20–22^ Among metal phosphates, alkali earth metal pyrophosphates (A_2_B_2_O_7_; A = Sr, Ba, Ca) have shown promise. Compared to phosphide and phosphate materials, pyrophosphate (P_2_O_7_^*n*–^)-*b*-based compounds are more stable and offer more significant space for ion traversal, which leads to faster electron transfer rates and better electrochemical properties.^20^ Over the past few decades, metal pyrophosphates have gained prominence due to their exceptional redox activities, broad operational potential window, natural abundance, remarkable reversibility, varied frameworks, structural integrity, and high electrical conductivity, rendering them highly beneficial for electrochemical studies.^23^ Particularly Sr_2_P_2_O_7_, which exists in orthorhombic and tetragonal phases, with the orthorhombic phase exhibiting enhanced electrochemical activity.^21,24,25^ Sr_2_P_2_O_7_’s applications in LEDs, bone ceramics, and catalysis demonstrate its potential in sustainable, cost-effective technologies.^23,26,27^ For instance, Sr_2_P_2_O_7_-based materials have been explored in LED phosphors and as catalysts for lactic acid dehydration to acrylic acid, further emphasizing their diverse applicability.^28,29^

In this study, we developed a modified screen-printed carbon electrode (SPCE) incorporating Sr_2_P_2_O_7_ for the electrochemical detection of synthetic VLN. Sr_2_P_2_O_7_ nanoparticles were synthesized via a facile solvothermal method, offering a robust and efficient route for metal phosphate production. Comprehensive characterization was performed using XRD, Raman, FT-IR, and FESEM techniques. The Sr_2_P_2_O_7_-modified electrode demonstrated notable electrocatalytic activity toward synthetic VLN, achieving high current responses, low detection limits, wide linear ranges, and high recovery rates. These findings suggest that Sr_2_P_2_O_7_ is a promising material for electrochemical applications, particularly in detecting synthetic VLN in food samples.

## Experimental Details

### Chemicals and Reagents

The materials utilized in this study included sodium dihydrogen phosphate (NaH_2_PO_4_ ≥ 98%), vanillin (C_8_H_8_O_3_ ≥ 99%), strontium nitrate (Sr­(NO_3_)_2_ ≥ 99%), sodium hydroxide (NaOH), potassium chloride (KCl ≥ 99%), sodium phosphate dibasic (Na_2_HPO_4_; ≥99.0%), ethanol, and acetone, all of which were procured from Sigma-Aldrich, Alfa Aesar, and Showa Chemical Industry Co., Ltd., and used without further purification. Ultrapure water (specific resistivity >18 MΩ·cm) obtained from a Milli-Q water purification system (Millipore, Molsheim, France) was employed in all experiments to ensure high purity standards. A 0.1 M phosphate buffer (PB) solution at pH 7, prepared using Na_2_HPO_4_ and NaH_22_PO_4_, was utilized as the supporting electrolyte across all electrochemical analyses.

### Synthesis of Strontium Phosphate

Using the solvothermal method, Kokulnathan et al.^20^ reported the synthesis of Sr_2_P_2_O_7_. According to their methodology, dihydrogen ammonium phosphate ((NH_4_)­H_2_PO_4_) and strontium nitrate (Sr­(NO_3_)_2_) were combined in a 2:1 molar ratio. The weighed precursors were then dissolved in deionized (DI) water and stirred at 80 °C for 3 h. After stirring, the resultant residue was isolated by centrifugation using a water and ethanol solution and subsequently dried in a hot air oven at 60 °C. The final white powder was calcined in a muffle furnace at 600 °C for 3 h to obtain Sr_2_P_2_O_7_, as shown in Figure 1a. The formation of Sr_2_P_2_O_7_ is described by the following reaction equation (eq 1).^20^

1 $${\mathrm{SrCO}}_{3}+2\left({\mathrm{NH}}_{4}\right)H_{2}{\mathrm{PO}}_{4}\to {\mathrm{Sr}}_{2}P_{2}O_{7}+2{\mathrm{NH}}_{3}+H_{2}O+3{\mathrm{CO}}_{2}$$

**Figure 1:**
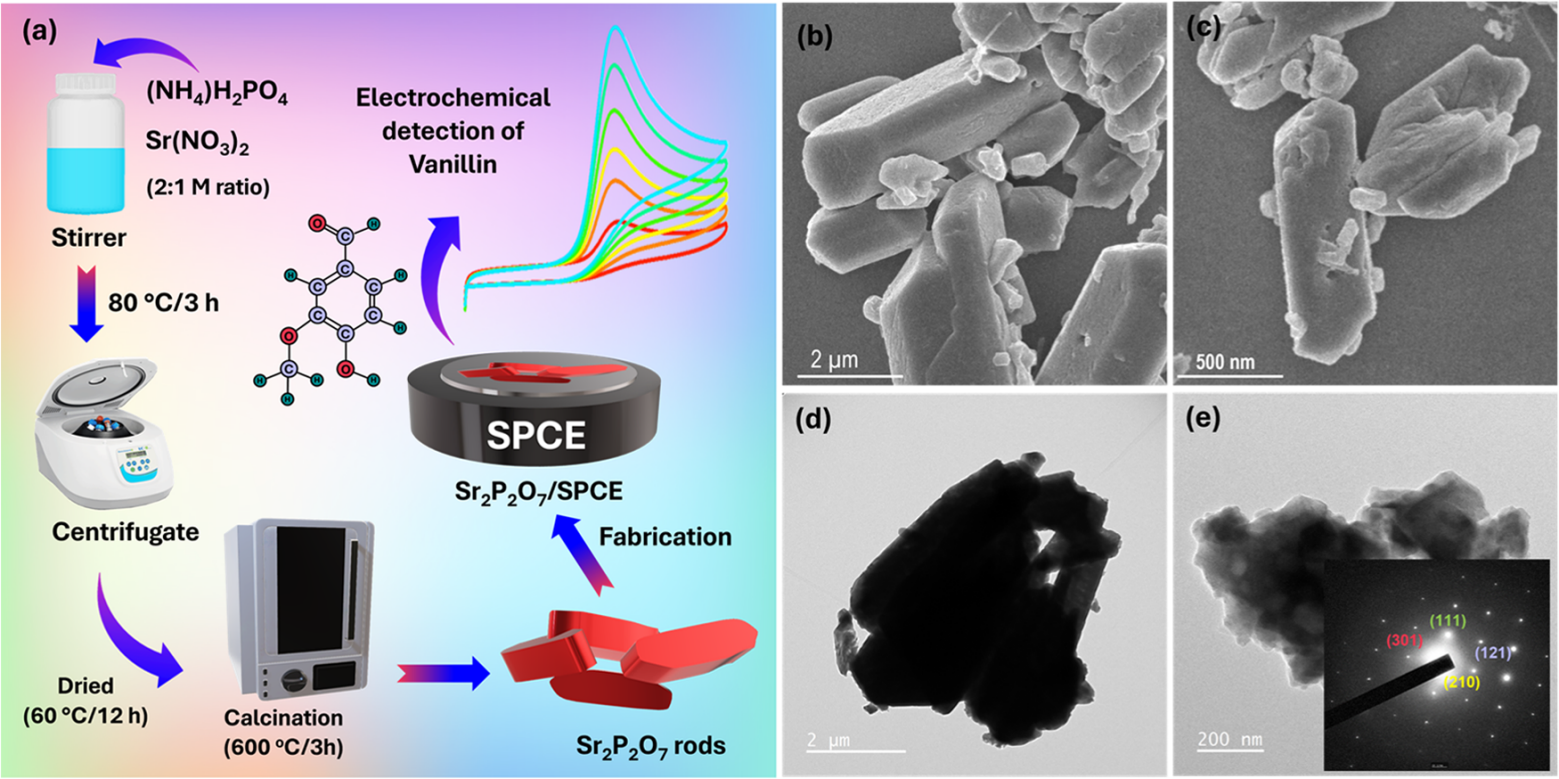
(a) Schematic illustration of synthesis and fabrication of Sr_2_P_2_O_7_ rods. (b, c) FESEM and (d, e) TEM of Sr_2_P_2_O_7_ rods along with its SAED patterns.

### Fabrication of Strontium Phosphate

Before the experiment, the SPCE surface was thoroughly precleaned with deionized (DI) water and ethanol solution, and then it was allowed to dry. Following this, 2 mg of Sr_2_P_2_O_7_ powder was mixed with 2 mL of DI water and sonicated for a few minutes to achieve complete dispersion. The resulting dispersed solution was then drop-cast onto the precleaned SPCE at an optimal loading amount of 6 μL/mg. Finally, the modified SPCE was dried in a hot air oven at 60 °C, preparing it for the electrochemical detection of synthetic VLN.

### Calculation of Average Crystalline Size (D)

The average crystallite size of Sr_2_P_2_O_7_ was calculated using the Scherrer equation (eq 2). In this equation, K represents the Scherrer constant, λ denotes the wavelength of the X-ray source, and β is the full width at half-maximum (fwhm) of the diffraction peak.^30^

2 D=Kλ/βcosθ

### Calculation of Electrochemical Active Surface Area (A)

The Randles–Sevcik equation (eq 3) can be used to determine the electroactive surface area of bare SPCE and Sr_2_P_2_O_7_/SPCE electrodes.^31–34^

3 $$I_{p}=2.69\times 10^{5}n^{3/2}AD_{0}^{1/2}Cv^{1/2}$$

Where, *n* is the number of electrons (*n* = 1), *I*_p_ is the peak current (*A*), *D*_0_ is the diffusion coefficient (cm^2^ s^–1^), *v* is the scan rate (V/s), *A* is the electroactive surface area (cm^2^), and *C* is the concentration of [Fe­(CN)_6_]^3–/4–^ (mol cm^–3^).^35^

### Calculation of the Limit of Detection

To determine the highest accuracy for sensitive detection of VLN, the amperometric (*i*–*t*) method was performed. For this, it utilizes 0.1 M phosphate-buffered (PB) as the electrolyte and sets the applied potential for the *i*–*t* measurement to 0.52 V in the three-electrode cell at the rotating disk electrode. eq 4 was used to determine the limit of detection (LOD):^34–37^

4 $$\mathrm{LOD}=\frac{3\mathrm{SD}}{m}$$

where *m* is the analytical sensitivity shown by the slope of the calibration plot, and SD is the standard deviation derived from three repeated measurements of the blank signal. The selectivity of Sr_2_P_2_O_7_/SPCE toward VLN is demonstrated by the *i*–*t* measurements when overlapping drugs are present.

## Results and Discussion

### Morphological Analysis

The surface morphologies of the synthesized Sr_2_P_2_O_7_ were examined using field emission scanning electron microscopy (FESEM) and transmission electron microscopy (TEM). The FESEM and TEM images of Sr_2_P_2_O_7_, presented in Figure 1b–e, reveal a rod-*like* structure. These Sr_2_P_2_O_7_ nanorods contribute to improved electrochemical stability. Additionally, the rough surface of the nanorods allows the analyte to penetrate more effectively, potentially enhancing the electrochemical sensing efficiency. Furthermore, the selected area electron diffraction (SAED) pattern (inset of Figure 1e) confirms the crystalline nature of Sr_2_P_2_O_7_, displaying diffraction planes corresponding to (301), (111), (121), and (210) orientations.

### Phase Analysis of Sr_2_P_2_O_7_

X-ray diffraction (XRD) examined the crystalline purity and structural characteristics of as-synthesized Sr_2_P_2_O_7_. Figure 2a shows well-defined sharp XRD pattern peaks corresponding to the hkl plans of (101), (002), (200), (103), (210), (013), (203), (301), (020), (105), (122), (401), (223), (315), (033). As-prepared material XRD peaks matched the JCPDS pattern of 01-087-0561 and exhibited orthorhombic crystal structure with space group 62. The space group and unit cell parameter of Sr_2_P_2_O_7_ is Pbnm and *a* = 8.94 Å, *b* = 5.41 Å, *c* = 13.21 Å, and the α = β = γ = 90.00. Based on the results, Sr_2_P_2_O_7_ formed with high crystallinity without any impurities. From eq 2, the calculated average crystalline value for Sr_2_P_2_O_7_ is ≈49 nm.

**Figure 2:**
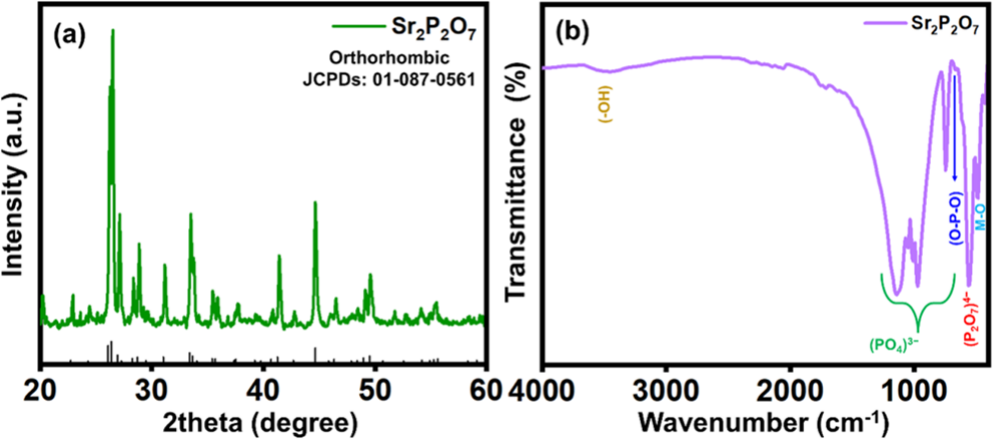
(a) XRD patterns and (b) FT-IR spectra of Sr_2_P_2_O_7_.

### Fourier Transform Infrared Spectroscopy (FT-IR) Analysis

Figure 2b provides structural insights into Sr_2_P_2_O_7_ particles, as observed through FT-IR spectroscopy conducted in the 400–4000 cm^–1^ region. The FT-IR spectrum of Sr_2_P_2_O_7_ exhibits bands consistent with previously reported functional groups, confirming the material’s structure. Notably, approximately 487 and 672 cm^–1^ characteristic bands correspond to pyrophosphate and metal–oxygen groups. The distinct band around 660 cm^–1^ is attributed to O–P–O bending vibrations, characteristic of the pyrophosphate structure. Symmetric and asymmetric stretching modes of (P–O) bonds appear as strong bands at approximately 872, 1070, and 1121 cm^–1^, indicating the presence of (PO_4_)^3–^ groups within the Sr_2_P_2_O_7_ structure. Additionally, a broad band above 2500 cm^–1^ is associated with adsorbed H_2_O on the surface of the Sr_2_P_2_O_7_.

### Electrocatalytic Activity of Modified Electrode

Electrochemical impedance spectroscopy (EIS) was conducted to assess the charge transfer resistance (*R*_ct_) at the electrode/electrolyte interface, driven by the sinusoidal electrochemical disturbance between the electrolyte and modified electrodes. The experiment was performed over a frequency range of 0.01 Hz to 100 kHz with an amplitude of 5 mV in a solution of 5 mM [Fe­(CN)_6_]^3–/4–^ and 0.1 M KCl. Figure 3a presents the Nyquist plot for various electrode configurations. The inset of Figure 3a illustrates the Randles circuit model, where C_dl_ denotes double-layer capacitance, *R*_ct_ indicates charge transfer resistance, *Z*_w_ represents Warburg impedance, and R_s_ corresponds to electrolyte resistance.^34–41^ *R*_ct_ is crucial for defining the resistance at the electrode/electrolyte interface, and in this study, it was found to be 1613.69 Ω·cm^2^ for bare SPCE and 365.68 Ω·cm^2^ for Sr_2_P_2_O_7_/SPCE, as illustrated in the bar diagram in Figure 3b. These findings indicate that the Sr_2_P_2_O_7_-modified SPCE exhibits a significantly lower *R*_ct_ compared to the bare SPCE, attributed to the material’s favorable properties, such as abundant active sites, enhanced electrical conductivity, efficient electron transport, and substantial active surface area.

**Figure 3:**
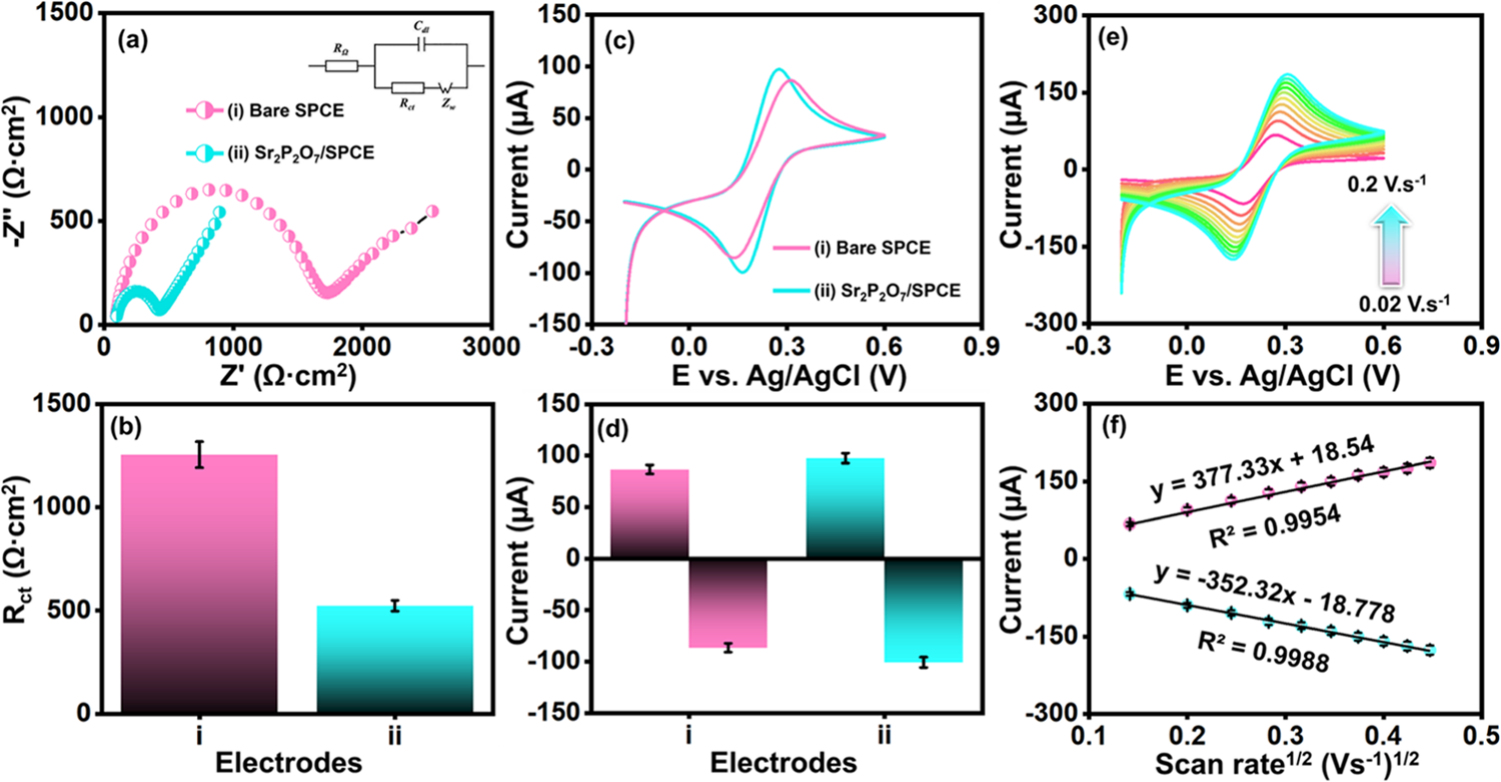
(a, b) EIS and (c, d) CVs of bare SPCE and Sr_2_P_2_O_7_ rod-modified SPCE in 0.1 M KCl and 5 mM of [Fe­(CN)_6_]^3‑,4‑^ solution and bar diagram of electrodes versus *R*_ct_ (Ω·cm^2^) and redox peak currents (μA). (e, f) CVs of Sr_2_P_2_O_7_/SPCE at various scan rate (0.02–0.2 V/s) and its linear plot for square root of the scan rate (V/s)^1/2^ versus redox peak current (μA).

To further investigate the electrode-to-electrolyte interaction, cyclic voltammetry (CV) was performed in the presence of 5 mM [Fe­(CN)_6_]^3–/4–^ with 0.1 M KCl. Figure 3c displays the CV responses of bare SPCE and Sr_2_P_2_O_7_/SPCE. The redox current values for the bare SPCE were measured as *I*_pa_ = 86.75 μA & *I*_pc_ = −86.41 μA at *E*_pa_ = 0.31 V & *E*_pc_ = 0.13 V, respectively. In contrast, Sr_2_P_2_O_7_/SPCE achieved redox current values of *I*_*p*a_ = 97.62 μA & *I*_pc_ = −100.67 μA at *E*_pa_ = 0.27 V & *E*_pc_ = 0.16 V, respectively. As shown in Figure 3d, the Sr_2_P_2_O_7_-modified SPCE exhibited higher peak currents and lower peak potentials compared to the bare SPCE. The peak separation (Δ*E*_p_) for the bare SPCE was 180 mV. In contrast, it was reduced to 110 mV for the Sr_2_P_2_O_7_-modified SPCE, further highlighting the favorable properties of Sr_2_P_2_O_7_, including active site abundance, high conductivity, efficient electron transfer, and substantial active surface area.

The CV technique was also applied to study the electrochemical electron transfer activity of Sr_2_P_2_O_7_/SPCE in 0.1 M KCl at different scan rates ranging from 0.02 to 0.2 V/s (Figure 3e). The redox peaks observed correspond to the oxidation of [Fe­(CN)_6_]^4–^ to [Fe­(CN)_6_]^3–^ and its reduction in the reverse process. The Sr_2_P_2_O_7_/SPCE displayed a linear increase in redox peak currents and a decreased peak-to-peak separation, demonstrating the efficient redox activity of the modified electrodes, as illustrated in Figure 3f. Linear regression and correlation coefficients were derived from these data, represented by equations in eqs 5 and 6.

**Oxidation:**

5 $$I_{pa}=377.33{\left(\frac{V}{s}\right)}^{1/2}+18.54;R^{2}=0.9954$$

**Reduction:**

6 $$I_{\mathrm{pc}}=-352.32{\left(\frac{V}{s}\right)}^{1/2}-18.778;R^{2}=0.9988$$

### Randles–Sevcik Equation

The Randles–Sevcik equation (eq 3) determined the electrochemically active surface area (EASA) of both bare SPCE and Sr_2_P_2_O_7_/SPCE. Figure 3c highlights the strong electrochemical activity of Sr_2_P_2_O_7_/SPCE, with linear regression plots showing high correlation coefficients *R* ^2^ = 0.9954 (*I*_pa_) and *R* ^2^ = 0.9988 (*I*_pc_), where the current response was plotted against the square root of the scan rate (Figure 3f). The electroactive surface area of Sr_2_P_2_O_7_/SPCE is calculated to be approximately 0.101 cm^2^, respectively, demonstrating a larger active surface area for the Sr_2_P_2_O_7_-modified electrode. This increase in EASA indicates that Sr_2_P_2_O_7_/SPCE has a more significant number of accessible active sites. The enhanced performance of Sr_2_P_2_O_7_/SPCE in electrochemical sensing is further supported by the following attributes: (i) The sheet-like structure of Sr_2_P_2_O_7_ provides ample active sites, beneficial for efficient VLN detection, and (ii) EIS measurements confirm that the rapid electron transport properties of Sr_2_P_2_O_7_/SPCE facilitate enhanced VLN detection capabilities.

### Electrochemical Activity of the Electrodes

The CV approach was employed to assess the electrochemical response of Sr_2_P_2_O_7_/SPCE in comparison with bare SPCE. The CV profiles of Sr_2_P_2_O_7_/SPCE and bare SPCE in the presence of 50 μM VLN in a 0.1 M phosphate buffer (PB) solution are presented in Figure 5a. The bare SPCE showed minimal peak response due to its limited electron transport capability and reduced active surface area. In contrast, the Sr_2_P_2_O_7_-modified SPCE exhibited a significantly enhanced oxidation peak, as shown in Figure 5b, attributed to the increased active sites and good electrical conductivity provided by the Sr_2_P_2_O_7_ modification. Compared to the bare SPCE, the Sr_2_P_2_O_7_/SPCE demonstrates a high active surface area and strong electron transport properties. This enhancement in oxidation peak current is further attributed to the interaction between the aromatic VLN and the modified electrode material facilitated by hydrogen bonding.

### Effect of Supporting Electrolyte

The optimization of the supporting electrolyte’s pH plays a crucial role in achieving highly sensitive and selective detection of the analyte. As shown in Figure 5c, (CV) curves of VLN on Sr_2_P_2_O_7_/SPCE were recorded across different pH values (3–11) at a fixed scan rate of 0.05 V/s. The results reveal that as the pH shifted from acidic to neutral, the oxidation peak current of VLN gradually increased, suggesting an optimal electron transfer rate between the electrode and electrolyte. The peak current reached its maximum in neutral media (pH 7) but decreased at alkaline pH values (9–11). This reduction in peak current at higher pH levels can be attributed to a higher concentration of negative charge carriers, which may create repulsive forces, hindering effective carrier interaction and reducing electron transfer efficiency. The bar diagram in Figure 5d further illustrates the relationship between oxidation peak current and pH. According to the Nernst equation, an equal number of electrons and protons participate in the electrochemical oxidation of VLN, affirming that pH 7 is the optimum condition for this reaction.

### Electrochemical Sensing Mechanism

Figure 4e illustrates the oxidation mechanism of VLN, which involves a two-electron transfer process. The mechanism of VLN oxidation on the working electrode surface is outlined in three stages. In the first stage, the VLN molecule diffuses toward the surface of the working electrode. During the second stage, oxidation occurs, converting VLN into its oxidized form, vanillic acid. This oxidation process involves the loss of two electrons, which are absorbed by the positively charged working electrode. In the final third stage, a reduction reaction produces vanillic acid as the end product. This stepwise mechanism, as shown in Figure 4e, highlights the electrochemical transformation of VLN and underscores the efficiency of the two-electron transfer process on the modified electrode surface (eqs 7–10).^3,4^

**Figure 4:**
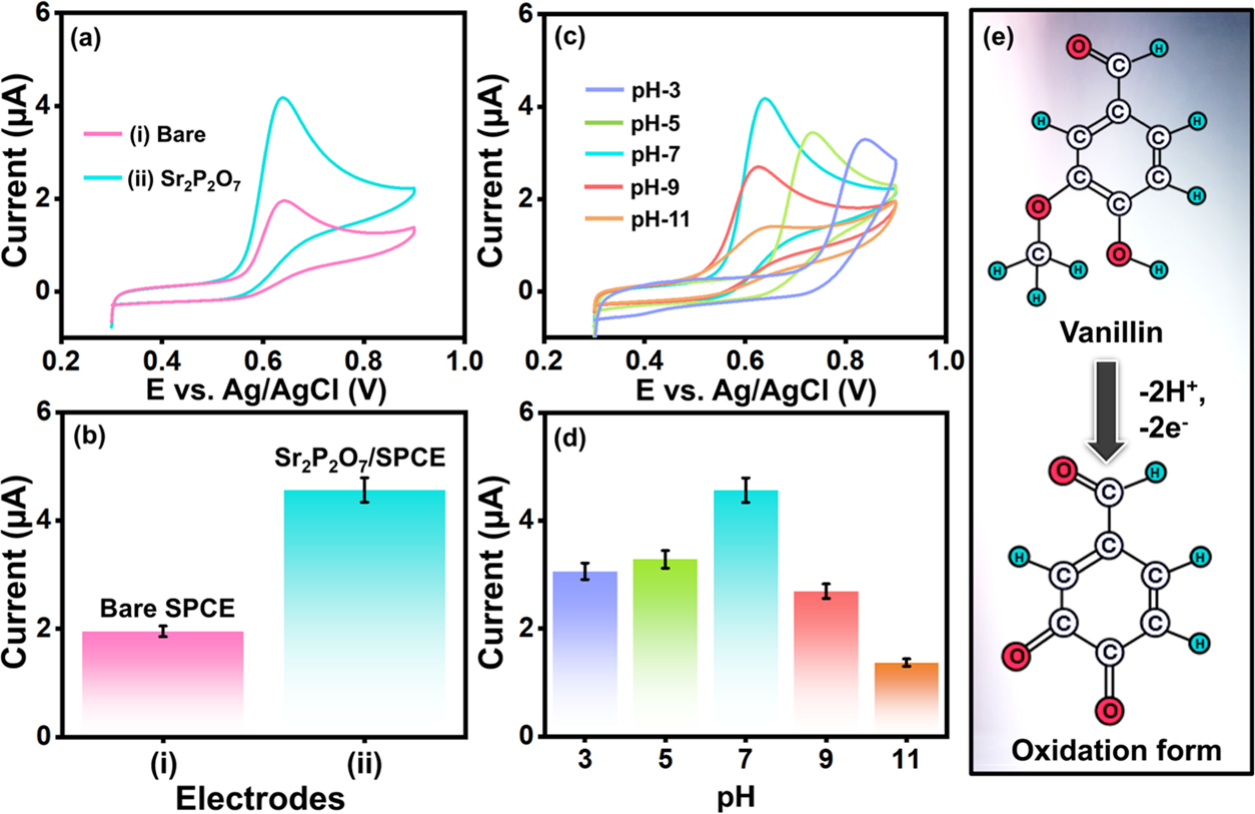
(a) CVs of bare SPCE and Sr_2_P_2_O_7_ nanorod-modified SPCE in the presence of VLN at 0.1 M PB and (b) bar diagram of electrodes versus peak currents (μA). (c) Effect of various supporting electrolytes (pH) at Sr_2_P_2_O_7_/SPCE in the presence of VLN at 0.1 M PB (pH-3 to pH-11) and (d) bar diagram of pHs versus peak currents (μA). (e) Possible electro-oxidation mechanism of VLN.

First Electron Transfer (–e^–^):

8 $$C_{6}H_{3}\left(\mathrm{OH}\right)\left({\mathrm{OCH}}_{3}\right)\left(\mathrm{CHO}\right)\to C_{6}H_{3}\left(O^{•}\right)\left({\mathrm{OCH}}_{3}\right)\left(\mathrm{CHO}\right)+e^{-}$$

Proton Loss (–H^+^):

9 $$C_{6}H_{3}\left(O^{•}\right)\left({\mathrm{OCH}}_{3}\right)\left(\mathrm{CHO}\right)\to C_{6}H_{3}\left(O^{-}\right)\left({\mathrm{OCH}}_{3}\right)\left(\mathrm{CHO}\right)+H^{+}$$

Second Electron Transfer (–e^–^):

10 $$C_{6}H_{3}\left(O^{-}\right)\left({\mathrm{OCH}}_{3}\right)\left(\mathrm{CHO}\right)\to C_{6}H_{3}\left(=O\right)\left({\mathrm{OCH}}_{3}\right)\left(\mathrm{CHO}\right)+e^{-}$$

Second Loss (–H^+^):

11 $$C_{6}H_{3}\left(=O\right)\left({\mathrm{OCH}}_{3}\right)\left(\mathrm{CHO}\right)\to C_{6}H_{2}\left(=O\right)\left({\mathrm{OCH}}_{3}\right)\left(\mathrm{CHO}\right)+H^{+}$$

### Influence of Catalyst Loading Amounts

The sensitivity of the electrode’s detection capability toward the analyte is influenced by the amount of catalyst loaded on the SPCE surface. This study explored various dosages (2–10 μL/mg) in a 0.1 M PB solution at a fixed scan rate of 0.05 V/s, as illustrated in Figure S1. As the dosage increased from 2 to 6 μL/mg, the oxidative peak current also increased, attributed to the active Sr_2_P_2_O_7_ nanorods facilitating electron transfer in the electrolyte solution. The peak current continued to rise at 8 μL/mg. Still, it then decreased at 10 μL/mg, likely due to excessive catalyst loading forming a thick layer on the SPCE surface. This obstructed pores and active sites, thereby hindering electron movement. Optimal loading of 6 μL/mg was selected for VLN detection, as it produced the highest oxidative peak current. This balance maximized electrochemical response by ensuring sufficient catalyst surface exposure without compromising electron mobility. The bar diagram in Figure S1 depicts the relationship between catalyst loading amount and VLN oxidative peak current.

### Effect of Concentration and Scan Rate

CV was conducted to analyze the response of Sr_2_P_2_O_7_-modified SPCE to varying VLN concentrations (20 to 120 μM) in a 0.1 M PB solution (pH 7) at a fixed scan rate of 0.05 V/s. Figure S2a shows that the oxidation peak current for VLN increased linearly with rising concentrations, indicating the rapid electron transport properties of the Sr_2_P_2_O_7_-modified electrode. This enhancement in peak current suggests that the Sr_2_P_2_O_7_/SPCE provides efficient electrolyte transport and facilitates fast electron transfer, thereby optimizing the electrochemical process. Furthermore, the consistent increase in peak current across concentrations demonstrates the antifouling capability of Sr_2_P_2_O_7_, maintaining effective sensing performance without degradation. The linear relationship between VLN concentration and oxidation peak current is depicted in Figure S2b. The linear regression and correlation coefficient values were derived from this linear plot (VLN concentrations vs peak current), as shown in eq 11.

12 $$I_{\mathrm{pa}}\left(\mu A\right)=0.0382\left[\mu M\right]+0.2932;R^{2}=0.9949$$

The kinetic behavior of VLN on Sr_2_P_2_O_7_/SPCE was examined at various scan rates using CV in a 0.1 M PB solution. Figure 5a displays the CV responses of Sr_2_P_2_O_7_/SPCE at scan rates ranging from 0.02 to 0.2 V/s for 50 μM VLN. The oxidation peak current increased linearly with rising scan rates, demonstrating that the scan speed influences the electrode response. This linear enhancement indicates that as the scan rate increases, the interaction time between the electrode surface and the electrolyte decreases, resulting in a slight shift in peak potential due to reduced interaction time.

**Figure 5:**
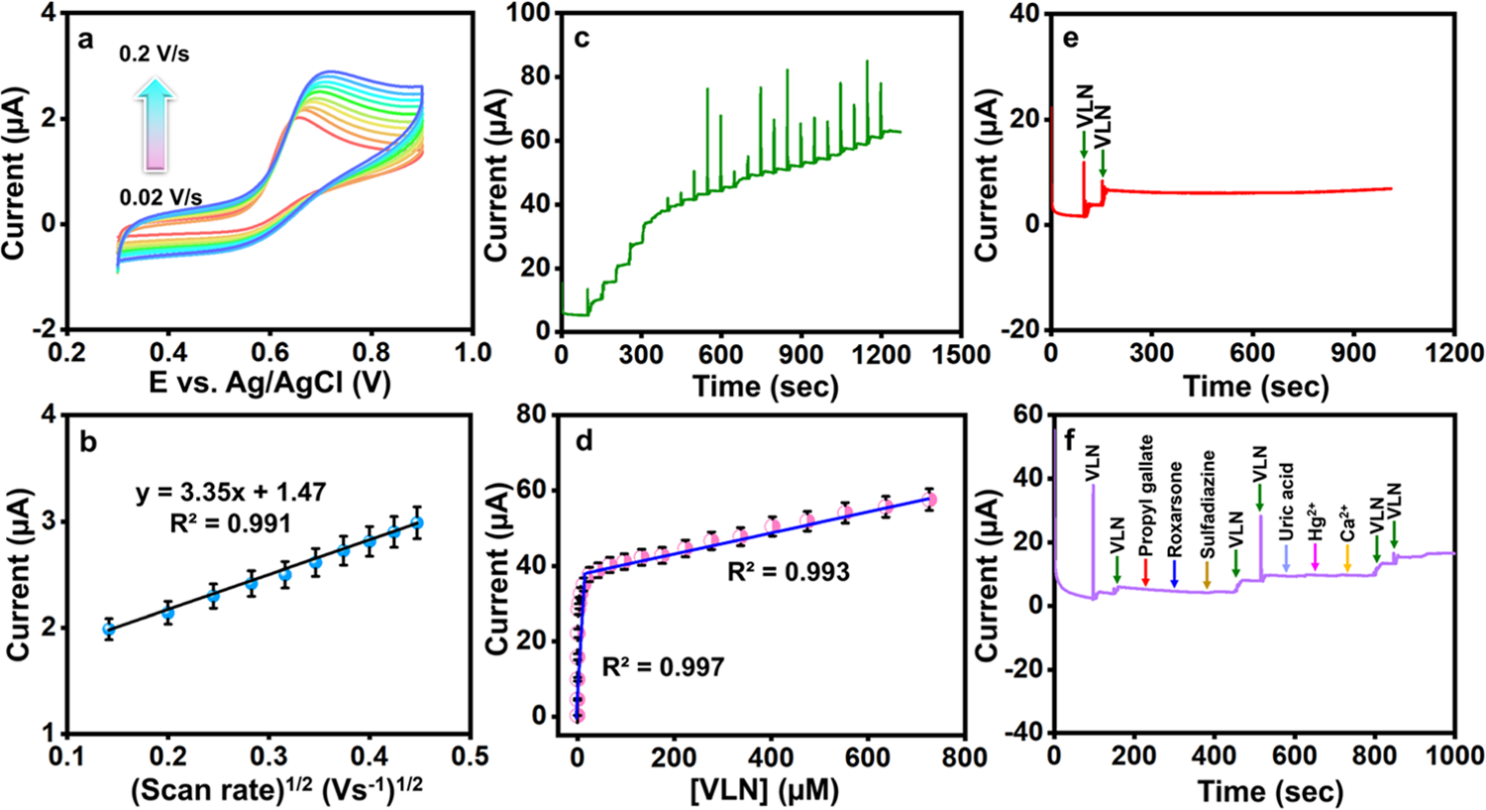
(a) CVs of Sr_2_P_2_O_7_/SPCE at various scan rates from 0.02 to 0.2 V/s with the presence of VLN in 0.1 M PB and (b) its linear plot for the square root of the scan rate (V/s)^1/2^ versus current (μA). (c) *i*–*t* responses Sr_2_P_2_O_7_ modified electrode at different concentrations (0.001–726.8 μM) of VLN at an applied potential of 0.52 V and rotation speed of 1200 rpm. (d) Corresponding linear plot for steady-state current (μA) versus VLN concentration (μM). (e) Operational stability of Sr_2_P_2_O_7_ modified electrode for 1000 s in the presence of VLN. (f) Selectivity of Sr_2_P_2_O_7_ modified electrode in the presence of 20 μM VLN and *5-fold* excess coexisting of potential interfering compounds.

At slower scan rates, there is ample time for interaction between the electrode and electrolyte, allowing for a stable *I*_pa_ response. The relationship between the square root of the scan rate and the peak current is illustrated in Figure 5b, where the linear regression and correlation coefficient values were obtained, as shown in eq 12. This relationship confirms the kinetic control of the electrochemical process. It validates the linear dependency of peak current on scan rate for Sr_2_P_2_O_7_/SPCE.

13 $$I_{\mathrm{pa}}\left(\mu A\right)=3.3597{\left(\frac{V}{s}\right)}^{1/2}+1.4715;R^{2}=0.9913$$

### Amperometric Analysis

The sensitive and selective technique of amperometry (*i*–*t*), operated at a single fixed potential, was employed for VLN detection, offering greater sensitivity and selectivity than differential pulse voltammetry (DPV) and cyclic voltammetry (CV). This method was used across various VLN concentrations to assess the performance of the Sr_2_P_2_O_7_-modified electrode. The experimental parameters were set as follows: potential at 0.52 V, rotation speed at 1200 rpm, and sensitivity at 1 × 10^–4^. VLN concentrations ranging from 0.001 to 726.8 μM were incrementally added to a 0.1 M PB solution at 50-s intervals. As illustrated in Figure 5c, the anodic peak current increased linearly with rising VLN concentrations, indicating effective detection capability. The calibration plot in Figure 5d shows the linear relationship between anodic peak current and VLN concentration, with two linear regions: one for lower concentrations (*R* ^2^ = 0.997) and another for higher concentrations (*R* ^2^ = 0.9934). The limit of detection (LOD) was calculated using eq 4 (LOD = 3SD/m), resulting in a remarkably low LOD of 0.52 nM within a linear range of 0.001–726.8 μM. This result highlights the high electrical conductivity and substantial surface area of the synthesized Sr_2_P_2_O_7_. Table S1 compares various modified electrodes used for VLN detection, including LODs and linear ranges reported in the literature, demonstrating the enhanced sensitivity of the Sr_2_P_2_O_7_-modified electrode in this study.

### Operational & Storage Stability, Selectivity, and Reproducibility Studies

The operational stability of the Sr_2_P_2_O_7_-modified electrode was evaluated using the amperometry (*i*–*t*) method over a period of 1000 s in a 0.1 M PB solution (pH 7) containing 10 μM VLN. As shown in Figure 5e, the peak current response remained stable, with minimal changes from the initial current, even after 1000 s, demonstrating the material’s stability during VLN detection. Additionally, analyzing VLN in food samples requires an assessment of selectivity due to the presence of potential interfering compounds. The anti-interference capability of the Sr_2_P_2_O_7_-modified electrode was tested using amperometry (*i*–*t*) against common cointerfering compounds (Figure 5f), including food additives, biomolecules, and metal ions. Interfering species such as propyl gallate, roxarsone, sulfadiazine, uric acid, Hg^2^ *^+^*, and Ca^2^ *^+^* were introduced at *5-fold* excess concentrations in a solution with 20 μM VLN. The results showed minimal impact on the current response, highlighting the electrode’s excellent selectivity for VLN detection. Furthermore, the reproducibility of the proposed sensor was verified using the CV method. The analysis was conducted in a 0.1 M PB solution with 100 μM VLN at a scan rate of 0.05 V/s, using four identically fabricated electrodes. The bar diagram in Figure S3 illustrates that the relative standard deviation (RSD) for VLN detection was below ±5%, confirming the reproducibility of the modified electrode material. The storage stability of the Sr_2_P_2_O_7_/SPCE was also assessed by storing the fabricated sensor for 15 days. In a 0.1 M PB solution with 100 μM VLN at a scan rate of 0.05 V/s, the RSD value was calculated as ±7% (Figure S4). These findings confirm that the Sr_2_P_2_O_7_-modified electrode exhibits good stability and reproducibility for VLN detection over time.

### Real Sample Analysis

Following the completion of all optimization procedures, the Sr_2_P_2_O_7_-modified electrode was applied to real-sample analysis. As discussed in the introduction, synthetic VLN is frequently found in various food products, such as ice cream and cake, with elevated levels posing potential health risks and environmental concerns. Thus, these two food samples were selected for quantitative analysis of VLN. In this approach, known amounts of VLN were spiked into prepared ice cream and cake samples prediluted to pH 7. Using the standard addition method, VLN was detected by amperometry (*i*–*t*) under the same optimized parameters as those established for VLN detection. Results demonstrated that the Sr_2_P_2_O_7_/SPCE sensor achieved an excellent recovery range of ±98.00–99.66% for both ice cream and cake samples, as shown in Table 1. These findings underscore the sensor’s high reliability and accuracy for detecting VLN in complex food matrices, confirming its suitability for practical applications in food safety monitoring.

**Table 1:** Recovery Range of Sr_2_P_2_O_7_-Modified Electrode at Various Real Samples

**sample**	**added (μM)**	**found (μM)**	**recovery %** (*n* = 3)
**cake**			
	3	2.98	99.33
	6	5.95	99.16
	9	8.91	99.00
**ice cream**			
	3	2.95	98.33
	6	5.88	98.00
	9	8.97	99.66

## Conclusions

In summary, the Sr_2_P_2_O_7_-modified electrode material was synthesized through a facile and efficient solvothermal method, proving to be a highly effective modifier that enhances the electrochemical performance of bare SPCE for VLN detection. The structural integrity and phase purity of Sr_2_P_2_O_7_ were confirmed by XRD and FT-IR. At the same time, TEM and FESEM provided detailed morphological insights, revealing a nanostructured material with abundant active sites. Electrochemical characterization through CV and amperometry (*i*–*t*) demonstrated that the Sr_2_P_2_O_7_-modified electrode exhibits enhanced catalytic activity, a broad linear detection range, and a remarkably low detection limit, indicating efficient electron transfer and high sensitivity. Real-sample analyses conducted with ice cream and cake showed satisfactory recovery rates, reinforcing the sensor’s reliability and selectivity in complex food matrices. Collectively, these results establish the Sr_2_P_2_O_7_-modified electrode as a robust and sensitive platform for the accurate detection of synthetic VLN in food products, highlighting its potential as a valuable tool for food safety and quality monitoring applications.

## Supplementary Material



## Notes

Detailed instrumentation and methods; Sr_2_P_2_O_7_ nanorods modified SPCE in the presence of VLN at catalyst various dosage level of (2–10 μL/mg) to SPCE (a) CV peaks of Sr_2_P_2_O_7_/SPCE at different concentrations. (b) Calibration plot of VLN concentrations (μM) vs peak current (μA); Comparison between various modified electrodes with Sr_2_P_2_O_7_ modified electrode; Reproducibility of Sr_2_P_2_O_7_/SPCE in the presence of 100 μM VLN in 0.1 M PB (pH 7); Days stability of Sr_2_P_2_O_7_/SPCE as its continuous usage for 15 days; Reference (PDF)
