# Supplementary material for: Electrochemical Detection of Synthetic Vanillin Using a Strontium Pyrophosphate Nanorod-Modified Electrode
Source: ACS Mater Au. 2025 Jan 21;5(4):632–40. doi: 10.1021/acsmaterialsau.4c00165 (PMC12257373; doi:10.1021/acsmaterialsau.4c00165)
Supplement: Supplementary file 1 [file mg4c00165_si_001.pdf]

Supporting information

Electrochemical Detection of Synthetic Vanillin Using Strontium Pyrophosphate Nanorods Modified Electrode

Balasubramanian Sriram[a], Sakthivel Kogularasu[b,c], Sea-Fue Wang[a,*], Guo-Ping Chang-Chien[b,c,d]

[a]Department of Materials and Mineral Resources Engineering, National Taipei University of Technology, Taipei 106, Taiwan.

[b]Super micro mass research and technology center, Cheng Shiu University, Kaohsiung 833301, Taiwan.

[c]Center for Environmental Toxin and Emerging-Contaminant Research, Cheng Shiu University, Kaohsiung 833301, Taiwan.

[d]Institute of Environmental Toxin and Emerging-Contaminant Research, Cheng Shiu University, Kaohsiung 833301, Taiwan.

*Corresponding author:

Dr. Sea-Fue Wang: sfwang@ntut.edu.tw

No. of Pages: 7

No. of Figures: 4

No. of Table: 1

Table of Content Entry		Page No.
Instrumentation and methods		S2
Figure S1	CVs of $Sr_2P_2O_7$ nanorods modified SPCE in the presence of VLN at catalyst various dosage level of (2-10 µL/mg) to SPCE in 0.1 M PB solution at fixed scan rate of 0.05 V/s.	S3
Figure S2	(a) CV peaks of $Sr_2P_2O_7$/SPCE at different concentrations from 20 to 120 µM. (b) Calibration plot of VLN concentrations (µM) vs. peak current (µA).	S3
Table S1	Comparison between various modified electrodes with $Sr_2P_2O_7$ modified electrode used for the detection of VLN.	S4
Figure S3	Reproducibility of $Sr_2P_2O_7$/SPCE in the presence of 100 µM VLN in 0.1 M PB (pH 7) for 4 different pack electrodes.	S5
Figure S4	Days stability of $Sr_2P_2O_7$/SPCE as its continuous usage for 15 days containing 100 µM VLN in 0.1 M pH 7.	S6
References		S6

Instrumentation and methods

To determine the phase configuration, X-ray diffraction (XRD) analysis was performed using a Bruker XRD D2 Phaser. Fourier transform infrared (FTIR) spectra were recorded with a Jasco FT-IR-4600 spectrophotometer. The surface morphology and elemental composition of the synthesized materials were examined through scanning electron microscopy (SEM, Hitachi S4700), high-resolution transmission electron microscopy (HR-TEM) (JEOL JEM-2100F, operating at 200 kV), and energy-dispersive X-ray spectroscopy (EDS) with EDAX AMETEK Inc. using DigitalMicrograph® software. These characterization techniques were essential for assessing the structural and compositional features of the synthesized materials. Electrochemical properties of the fabricated materials were investigated with a Metrohm Autolab (AUT51770, 100-240V~75VA 50/60Hz), which provided insights into the charge transfer resistance. Electrochemical measurements, including cyclic voltammetry (CV) and amperometry (i-t), were conducted in a conventional three-electrode system using a CHI 1211C electrocatalytic workstation. In this setup, a saturated Ag|AgCl electrode, platinum (Pt) wire, and a screen-printed carbon electrode (SPCE) (surface area = 0.071 cm^2) served as the reference, counter, and working electrodes, respectively.

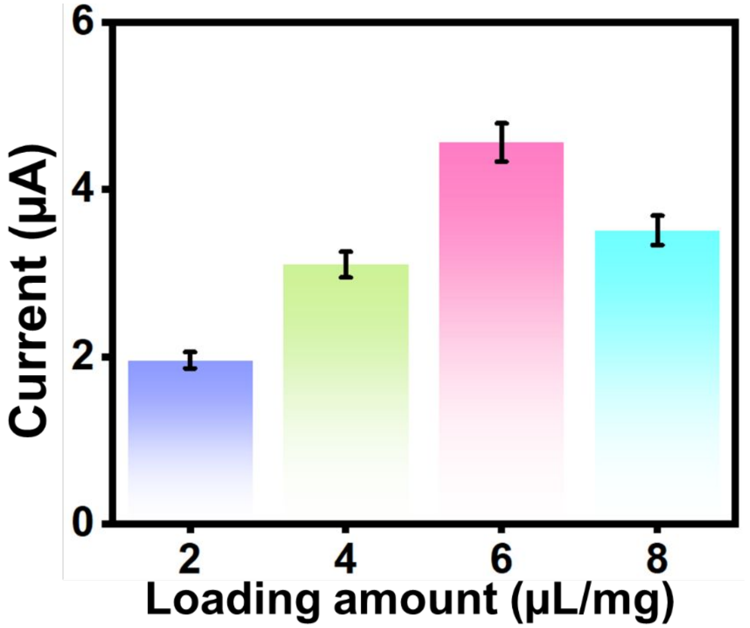

Figure S1. $Sr_2P_2O_7$ nanorods modified SPCE in the presence of VLN at catalyst various dosage level of (2-10 µL/mg) to SPCE in 0.1 M PB solution at fixed scan rate of 0.05 V/s.

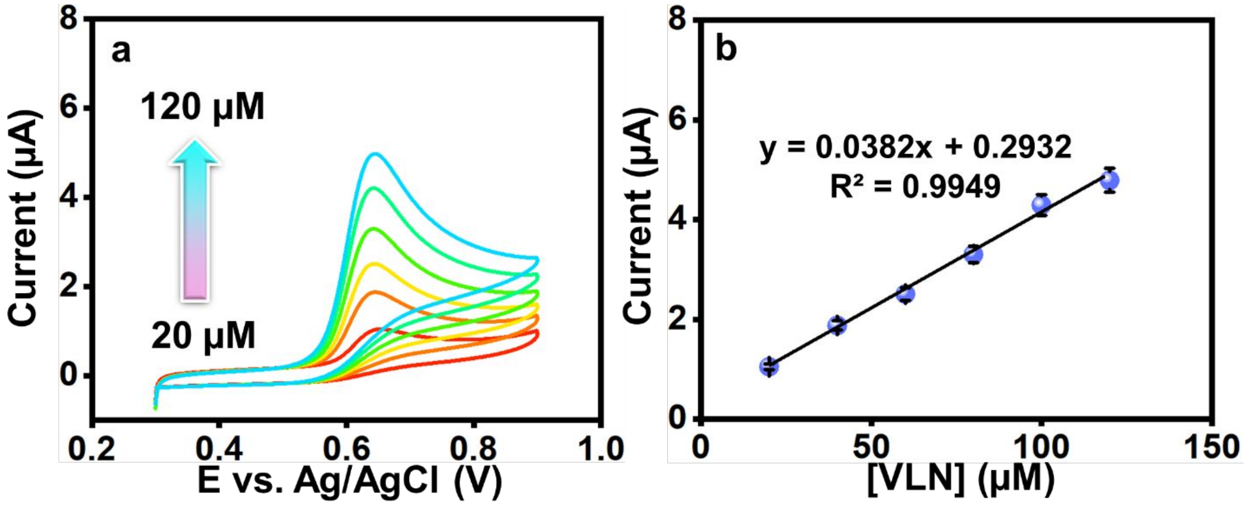

Figure S2. (**a**) CV peaks of $Sr_2P_2O_7$/SPCE at different concentrations from 20 to 120 µM. (**b**) Calibration plot of VLN concentrations (µM) vs. peak current (µA).

Table S1. Comparison between various modified electrodes with $Sr_2P_2O_7$ modified electrode used for the detection of VLN.

Materials	Methods	Linear range (µM)	LOD (nM)	Ref.
Ar[a]–Gr[b]/GCE[c]	DPV	2-70	1000	S1
$Fe@Fe_3C$–C	DPV	0.01–50	2.6	S2
GCE	SWV	50–300	160	S3
ENGR[d]–CNTs[e]	SWV[i]	0.01–10	20	S4
TBAC-900	LSV	5–1150	680	S5
Gr–PVP[f]/ABPE[g]	DV[j]	0.02–100	10	S6
AuPd–Gr/GCE	DPV	0.1–7; 10–40	20	S7
Gr/GCE	DPV	0.6–48	50	S8
ZnCr-LDH@g-CN	DPV	0.001–143.2	0.9	S9
**$Sr_2P_2O_7$/SPCE**	**i-t**	**0.001–726.8**	**0.52**	**This work**

[a]Arginine. [b]Graphene. [c]Glassy carbon electrode. [d]Nitrogen-doped graphene. [e]Carbon nanotubes. [f]Polyvinylpyrrolidone. [g]Acetylene blackpasteelectrode. [h]Differential pulse voltammetry. [i] Square wave voltammetry. [j]Derivative voltammetry.

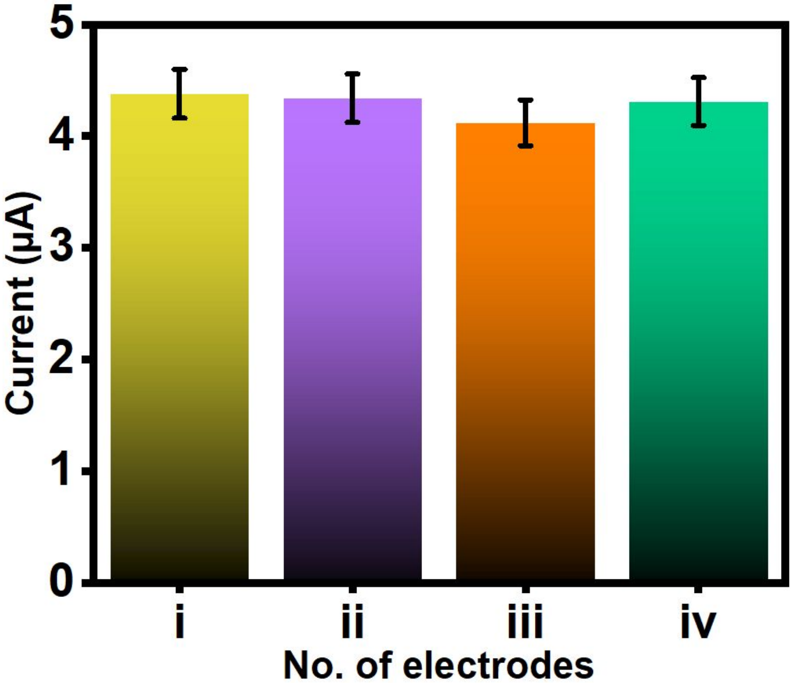

Figure S3. Reproducibility of $Sr_2P_2O_7$/SPCE in the presence of 100 µM VLN in 0.1 M PB (pH 7) for 4 different pack electrodes.

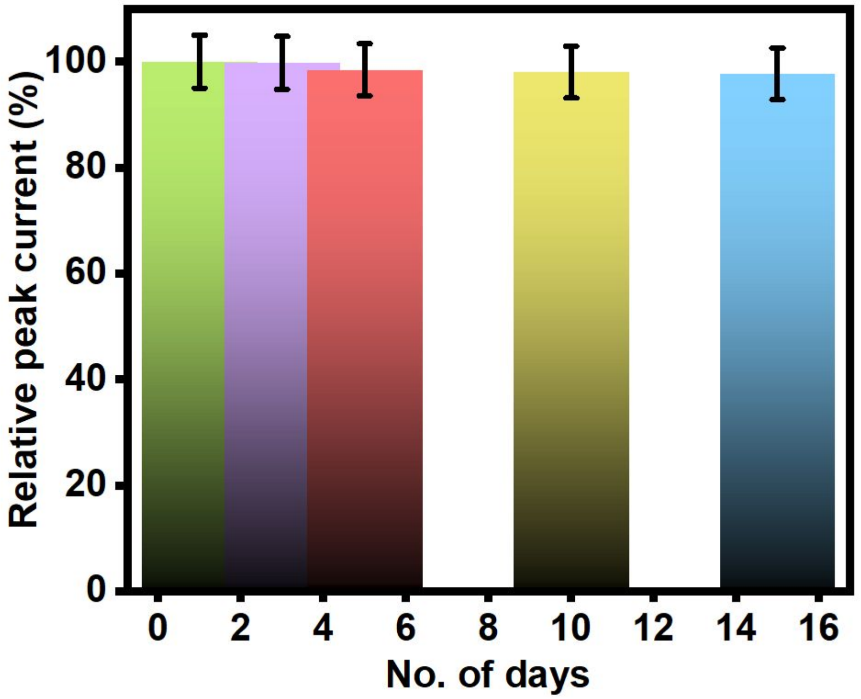

Figure S4. Days stability of $Sr_2P_2O_7$/SPCE as its continuous usage for 15 days containing 100 µM VLN in 0.1 M pH 7.

Reference:

S1. Zhao, Y., Du, Y., Lu, D., Wang, L., Ma, D., Ju, T. and Wu, M., 2014. Sensitive determination of vanillin based on an arginine functionalized graphene film. *Analytical Methods*, *6*(6), pp.1753-1758.

S2. Sun, J., Gan, T., Wang, K., Shi, Z., Li, J. and Wang, L., 2014. A novel sensing platform based on a core–shell Fe@Fe_3C–C nanocomposite for ultrasensitive determination of vanillin. *Analytical Methods*, *6*(15), pp.5639-5646.

S3. Hardcastle, J.L., Paterson, C.J. and Compton, R.G., 2001. Biphasic sonoelectroanalysis: Simultaneous extraction from, and determination of vanillin in food flavoring. *Electroanalysis: An International Journal Devoted to Fundamental and Practical Aspects of Electroanalysis*, *13*(11), pp.899-905.

S4. Luo, S. and Liu, Y., 2012. Poly (acid chrome blue K) modified glassy carbon electrode for the determination of vanillin. *International Journal of Electrochemical Science*, *7*(7), pp.6396-6405.

S5. Veeramani, V., Madhu, R., Chen, S.M., Veerakumar, P., Syu, J.J. and Liu, S.B., 2015. Cajeput tree bark derived activated carbon for the practical electrochemical detection of vanillin. *New Journal of Chemistry*, *39*(12), pp.9109-9115.
S6. Deng, P., Xu, Z., Zeng, R. and Ding, C., 2015. Electrochemical behavior and voltammetric determination of vanillin based on an acetylene black paste electrode modified with graphene–polyvinylpyrrolidone composite film. *Food chemistry*, *180*, pp.156-163.
S7. Shang, L., Zhao, F. and Zeng, B., 2014. Sensitive voltammetric determination of vanillin with an AuPd nanoparticles− graphene composite modified electrode. *Food chemistry*, *151*, pp.53-57.
S8. Peng, J., Hou, C. and Hu, X., 2012. A graphene-based electrochemical sensor for sensitive detection of vanillin. *International Journal of Electrochemical Science*, *7*(2), pp.1724-1733.
S9. Gopi, S. and Wang, S.F., 2023. Electrochemical determination of vanillin using 2D/2D heterostructure based on ZnCr-layered double hydroxide and g-CN. *Microchimica Acta*, *190*(10), p.423.
